# Bone Regeneration in Allergic Fungal Rhinosinusitis: Post-treatment Image Follow Up

**DOI:** 10.7759/cureus.6502

**Published:** 2019-12-29

**Authors:** Ibrahim Alarifi, Surayie Al-Dousary, Amal Bin Hazza’a, Ibrahim Sumaily

**Affiliations:** 1 Otorhinolaryngology, Security Forces Hospital, Riyadh, SAU; 2 Otorhinolaryngology, King Saud University, King Abdulaziz University Hospital, Riyadh, SAU; 3 Otorhinolaryngology, King Abdulaziz University Hospital, Riyadh, SAU

**Keywords:** allergic fungal rhinosinusitis, fungi, allergy, multidetector computed tomography, paranasal sinus, bone erosion score, bone regeneration, bone healing

## Abstract

Background: Bone erosion is a feature of allergic fungal rhinosinusitis (AFRS). The incidence of bone regeneration after treatment is unknown. The objective of this study is to evaluate the fate of bone erosion in AFRS.

Materials and methods: A retrospective chart review was conducted for all AFRS patients who had preoperative evidence of bone erosion on the computed tomography (CT) of the paranasal sinus (PNS) and at least one postoperative image. We used the bone erosion score (BES) as a valid tool to evaluate the preoperative bone erosion extension and the rate of regeneration on postoperative images.

Results: A total of 40 patients met our enrollment criteria. Of these, 30 patients underwent CT within nine months postoperatively (mean baseline BES: 9.07, SD: 8.11), 33 patients underwent CT between nine and 18 months postoperatively (mean baseline BES: 9.36, SD: 8.80), and 34 patients underwent CT after 18 months postoperatively (mean baseline BES: 8.56, SD: 7.69). The BES improved by 88.92% within nine months postoperatively with a follow-up BES of 0.93 (SD: 1.65), by 90.29% between nine and 18 months with a follow-up BES of 0.91 (SD: 2.15), and by 96.57% after 18 months with a follow-up BES of 0.29 (SD: 0.62). Complete bone regeneration occurred in 27 patients (67.50%); this was noted within nine months in 21 patients and after 18 months in six. Among them, 16 were male and 11 were female (p = 0.75). Regarding their ages, eight were pediatric and 19 were adult patients (p = 0.31).

Conclusion: Bone erosion in AFRS is a mostly reversible process. Complete bone regeneration occurred in more than two-thirds of patients within a short period of time.

## Introduction

Bone erosion is commonly seen in allergic fungal rhinosinusitis (AFRS) patients. It is usually seen as an expansion and loss of continuation of the paranasal sinus (PNS) wall on a computed tomography (CT) image [1]. AFRS is a disease of immunocompetent susceptible individuals [2-3]. Bent et al. described the diagnostic criteria for AFRS, which became known as the Bent and Kuhn criteria for AFRS diagnosis. They also described other less common findings, including bone erosion of PNS walls [4].

Bone erosion of the PNS walls has significant clinical consequences, such as intraorbital or intracranial disease extension, and may lead to ophthalmological or neurological sequelae [5-6]. Transient and even permanent visual loss may result as a complication of bone erosion of the sphenoid sinus wall and subsequent optic nerve compression or neuritis [7-8]. Despite the important consequences of bone erosion, no previous studies have followed up patients with AFRS-related bone erosion and its regeneration rate in the long term. Therefore, the aim of this study is to evaluate the fate of bone erosion in AFRS cases using a validated scoring tool.

## Materials and methods

A retrospective chart review was performed for all patients who fulfilled the Bent and Kuhn criteria for AFRS diagnosis in the period between March 2008 and January 2018 at King Abdulaziz University Hospital, King Saud University, Riyadh, Saudi Arabia. All patients who had radiographic evidence of bone erosion on the preoperative non-contrasted thin-cut CT of PNS and had at least one postoperative follow-up image were included. We excluded patients with a diagnosis of bone disorders, history of major facial trauma and fractures, or iatrogenic bone injury. We also excluded any patient with evidence of disease recurrence on post-treatment follow-up CT. Demographic data, including age at diagnosis and sex, were collected for all cases.

As a valid tool to evaluate the degree of bone erosion and its regeneration, we used the scoring system proposed by Al-Dousary et al. [1]. Based on the proportion of the PNS wall that was eroded and expanded, the erosion was graded from 1 to 3 (1 if less than one-third, 2 if between one-third and two-thirds, and 3 if more than two-thirds of the wall was eroded). The bone erosion score (BES) was calculated as the sum of the grades of bone erosion in the clinically relevant PNS walls and ranged from 0 to 72. We reviewed all the postoperative follow-up CT images to detect the degree of bone regeneration at different points in time. We calculated the BES for each of these images and compared it with the preoperative score to detect bone regeneration and evaluate its extent. The standard management of AFRS at our institute consists of endoscopic sinus surgery combined with post-operative oral and intranasal steroid treatment. This management protocol is consistent with the literature [9].

The results are expressed as the mean, standard deviation (SD), and confidence interval (CI) at the 90% confidence level for continuous variables (age and BES) and as the number (n) and percentage for categorical variables (patient sex). The data were analyzed using Statistical Package for the Social Sciences (SPSS); version 23.0 software (IBM Corp., Armonk, NY, USA).

## Results

A total of 40 patients with a diagnosis of AFRS met our eligibility criteria. Of these, 23 (57.5%) were male and 17 (42.5%) were female. Regarding age, 14 were pediatric and 26 were adult patients. The mean patient age at diagnosis was 23.6 years (SD: 8.58). The mean preoperative baseline BES was 9.30 (SD: 8.44; 90% CI: 7.10-11.50).

Out of the 40 AFRS patients, 30 patients underwent CT within nine months postoperatively (mean baseline BES: 9.07, SD: 8.11; 90% CI: 6.61-11.53), 33 patients underwent CT between nine and 18 months postoperatively (mean baseline BES: 9.36, SD: 8.80; 90% CI: 6.84-11.88), and 34 patients underwent CT after 18 months postoperatively (mean baseline BES: 8.56, SD: 7.69; 90% CI: 6.40-10.73). The BES improved by 88.92% within nine months postoperatively (mean follow-up BES: 0.93, SD: 1.65; 90% CI: 0.43-1.43), 90.29% between nine and 18 months postoperatively (mean follow-up BES: 0.91, SD 2.15; 90% CI: 0.29-1.53), and 96.57% after 18 months postoperatively (mean follow-up BES: 0.29, SD: 0.62; 90% CI: 0.12-0.46) (Table 1).

**Table 1:** Bone erosion scores during postoperative follow-up in 40 patients with allergic fungal rhinosinusitis SD: standard deviation.

Bone Erosion Score	Postoperative Follow-up Image Time		
	< 9 Months (n = 30)	9–18 Months (n = 33)	> 18 Months (n = 34)
Baseline Mean (SD)	9.07 (8.10)	9.36 (8.80)	8.56 (7.69)
Follow-up Mean (SD)	0.93 (1.65)	0.91 (2.15)	0.29 (0.62)
Improvement (%)	88.92%	90.29%	96.57%

**Figure 1:**
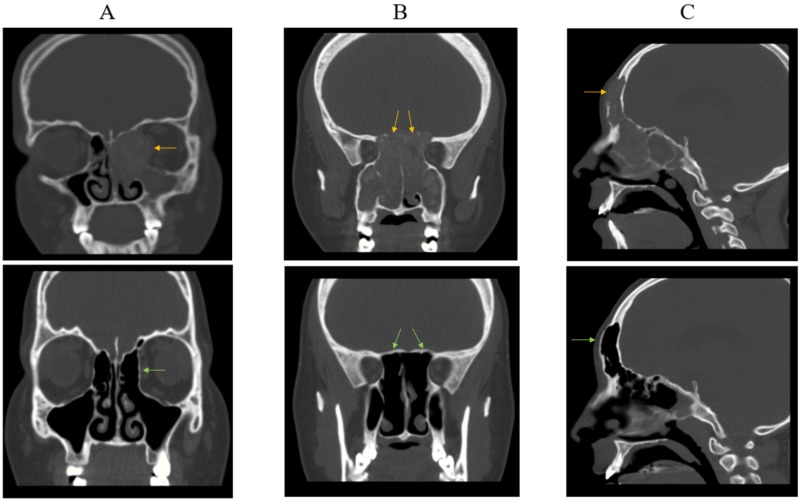
Computed tomography images for three patients showing improvement in erosion grade and the overall calculated bone erosion score (BES) (A) Upper panel: Grade 3 erosion of the lamina papyracea at the left anterior ethmoid sinus with an overall BES of 12. Lower panel: a postoperative image after 18 months showing no erosion with a BES of 0. (B) Upper panel: Grade 3 erosion of the roof of the posterior ethmoid sinus on both sides with a BES of 32. Lower panel: a postoperative image at nine months showing no erosion and a BES of 0. (C) Upper panel: Grade 2 erosion of the right anterior frontal table with a BES of 20. Lower panel: a postoperative image at nine months showing no erosion and improvement in the overall BES to 5, with some residual erosion at other sinuses (not shown).

Complete bone regeneration occurred in 27 patients (67.50%); this was noted within nine months in 21 patients and after 18 months in six. Among them, 16 were male and 11 were female (p = 0.75). Regarding their ages, eight were pediatric and 19 were adult patients (p = 0.31). Table 2 compares the patients with complete bone regeneration to those with incomplete regeneration. Examples of the improvement in bone erosion grades and overall BES are shown in Figure 1.

**Table 2:** Demographic characteristics of AFRS patients with postoperative complete bone regeneration compared to those with incomplete bone regeneration as evidenced by computed tomography AFRS: allergic fungal rhinosinusitis.

Characteristics		Complete Regeneration (n = 27)	Incomplete Regeneration (n = 13)	P Value
Sex	Male (n = 23)	16	7	0.75
	Female (n = 17)	11	6	
Age	Adult (n = 26)	19	7	0.31
	Pediatric (n = 14)	8	6	

## Discussion

The main finding of this study is that complete bone regeneration occurred in more than two-thirds of patients with AFRS-related bone erosion. Across the literature, the rate of bone erosion in AFRS has been thoroughly studied and ranges from 20% to 58% [1,10-16]. AFRS is a non-invasive fungal sinusitis, and the process of bone erosion is usually attributed to pressure atrophy and inflammatory mediators induced by the accumulated fungal debris. However, PNS mucosa and periosteum are usually intact [5,17-18]. Bone loss of the sphenoid wall may lead to multiple cranial neuropathies as a result of the pressure of fungal debris on the surrounding neuronal structures [18].

To the best of our knowledge, this is the first study to assess the rate of bone regeneration in AFRS patients. We utilized BES to evaluate the rate of bone regeneration over time. BES is a valid tool introduced recently by Al-Dousary et al. [1] to assess the degree of bone erosion in AFRS patients. In our sample, the BES improved by almost 90% within nine months of postoperative follow-up. Complete bone regeneration occurred in more than half of the patients within the same time period. This indicates that bone erosion in AFRS is a rapidly reversible process. In this study, the rate of bone regeneration was not affected by the patients’ sex or age. Almost one-third of our sample were pediatric patients, and they showed no difference in the rate of bone regeneration compared to adult patients.

We showed the valuable usage of BES in the assessment of bone regeneration after treatment of AFRS. BES was introduced previously by Al-Dousary et al. [1] as an objective tool for assessing the degree of bone erosion and its severity in AFRS cases. Furthermore, it may assist in surgical planning, such as the duration of surgery, patient counseling, and anesthesia considerations. We anticipate that the BES will be an important tool in future studies and will facilitate communication between researchers and clinicians when assessing the severity of bone erosion and degree of regeneration in patients with AFRS.

A major limitation of this study is its retrospective design. All patients underwent endoscopic sinus surgery as the mainstay of treatment with no intraoperative complications recorded. However, the postoperative treatment duration was variable and depended on the individual case and the surgeons’ preferences. Iatrogenic injury of the PNS walls during surgery could not be determined because of missing information in the medical records. Nevertheless, the post-operative BES improved significantly. A prospective long-term study could overcome this limitation but might be difficult to conduct because of the relative rarity of this disease entity.

## Conclusions

This study is the first to evaluate the fate of bone erosion in patients with AFRS. Bone erosion in AFRS is a mostly reversible process. In this study, complete bone regeneration occurred in more than two-thirds of patients within a short period of time, and the remaining one-third continued to have minimal residual erosion.
